# A well-being programme in severe mental illness. Baseline findings in a UK cohort

**DOI:** 10.1111/j.1742-1241.2007.01605.x

**Published:** 2007-12

**Authors:** S Smith, D Yeomans, C J P Bushe, C Eriksson, T Harrison, R Holmes, L Mynors-Wallis, H Oatway, G Sullivan

**Affiliations:** 1 Institute of PsychiatryCamberwell, London, UK; 2 Leeds Partnerships Foundation NHS TrustLeeds, UK; 3 Eli Lilly and Company Ltd, Lilly HouseBasingstoke, UK; 4 Scarborough HouseSparkbrook, Birmingham, UK; 5 Caludon CentreCoventry, UK; 6 Alderney HospitalPoole, UK; 7 Teesbay Unit, St Lukes HospitalMiddlesbrough, UK; 8 School of Care Sciences, University of GlamorganMerthyr Tydfil, UK

## Abstract

**Introduction:**

Patients with severe mental illness (SMI) have higher rates of cardiovascular disease (CVD) morbidity and mortality than the general population. In the UK, data were limited regarding the known prevalence of physical health screening of SMI patients.

**Aims:**

A total of 966 patients with SMI from seven geographically varied regions in the UK agreed to participate in a 2-year nurse-led intervention (Well-being Support Programme), designed to improve their overall physical health by providing basic physical health checks, health promotion advice, weight management and physical activity groups in secondary care.

**Results:**

At baseline, only 31% of participants had undergone a recent physical health check. There were high rates of obesity (BMI > 30 in 49%), glucose abnormalities (12.4%), hypertension/prehypertension (50%), hyperlipidaemia (71%), poor diet (32%), low exercise levels (37.4%) and smoking (50%).

**Conclusions:**

Patients with SMI where healthcare professionals have concerns regarding their physical health, have potentially modifiable risk factors for CVD, which remain undiagnosed. Programmes designed to address the physical health problems in SMI need to be implemented and evaluated in this already marginalised group of people.

**What's known**

Severe mental illness (SMI) is associated with a variety of medical illness found in excess in comparison to a general population. Much of these physical illness data is derived from outside UK and is focussed on selected cardiovascular risk factors.

**What's new**

Most SMI patients in the UK have risk factors for significant physical illness including overweight, smoking, low activity levels and poor diet. The provision of physical health checks varies across the UK with an average of 31% SMI patients getting a yearly review. SMI patients need targeted physical health programmes to address common modifiable risk factors for avoidable morbidity and early death.

## Introduction

Having a severe mental illness (SMI) is associated with having compromised physical health. Although SMI can be regarded as an imprecise diagnosis the concept of SMI is a clinical one and is included in many government policy documents and other bodies. Life expectancy in schizophrenia is 20% lower than in the general population (1) and two-thirds of the excess mortality seen in the schizophrenia patient population is attributable to natural (predominantly cardiovascular and respiratory) causes (2,3). Intrinsic risk factors, such as age, familial traits or ethnicity, increase an individual's vulnerability to physical illness, but it is also evident that people with SMI are more likely to have lifestyles which increase their risk of preventable physical disease (4–6). Such lifestyle factors include smoking, obesity, poor diet, lack of exercise and poverty (4,5,7,8). Some of the excess mortality seen in SMI might be reduced if attention were paid to these modifiable risk factors.

Evidence suggests that these risk factors for physical illness are not routinely measured in this population in the UK. A review of screening for dyslipidaemia in 606 patients in UK found that lipid screening had been undertaken in only 3.5% of patients with SMI taking antipsychotics (9). Furthermore there appear to be very few services designed to tackle the high level of physical health problems seen in this group. Patients with SMI may not proactively attend their GP for healthcare and such healthcare is not always proactively offered by GPs. Kendrick (10) found that despite presenting to primary care services three to four times more frequently than the general population, physical health risk factors in patients with SMI were rarely monitored by GPs. Thus, despite the recognition that there is an increased prevalence of risk factors for cardiovascular disease (CVD) in this population, there is little routine recording of blood pressure, weight, glucose or lipid screening, either in primary or secondary care services. Not much evidence exists of services that might provide smoking cessation or dietary improvements. However, the evidence suggests that when health screening from a GP is offered patients will accept. A recent UK study reported that a similar number of patients with psychosis accepted a postal invitation to undergo screening for cardiovascular risk factors as non-psychotic patients (11).

The overall evidence from a complete review of lifestyle management studies is that most patients will accept entry into an appropriate programme (8). There are clear benefits of physical health interventions being available at any point of contact with a healthcare professional, not only with the GP. People with SMI are often in regular contact with a mental health team, yet many do not have regular contact with primary care services.

Any physical health improvement intervention should target not only weight but also diet, smoking, exercise levels, dietary choices and be primarily educational and long term. The issue is not so much whether it should be done or who should be responsible for delivering it, but when and how and the optimum type of programme.

The Well-Being Support programme in the UK has been running for 2 years. The UK government recently endorsed this programme and its aims (12). This paper focusses on the demographics and the degree of baseline physical pathology in a large geographically diverse group of patients with SMI, who entered the WSP due to healthcare professional concern regarding their physical health monitoring.

## Method

A total of 966 outpatients with SMI (> 2 years) were enrolled in the WSP. All patients who joined the programme signed consent forms agreeing to basic physical health checks, lifestyle advice and if appropriate to participate in healthy living groups. They also consented to anonymised data being used to form the basis of an evaluation of this service. Ethical approval was not sought as the WSP is not an intervention as such but provided a service relevant for SMI patients in secondary care. Such care for non-mentally ill subjects would usually be provided in primary care.

### WSP implementation

Seven Mental Health Trusts from around England and Wales took part in the service, encompassing a wide geographical and socio-economic spread, from inner city and rural-deprived areas to more affluent suburban areas. At each centre, a registered mental nurse, trained in monitoring the physical health of people with SMI worked with a lead psychiatric consultant. Full details of WSP have previously been published (13). In summary, a register of patients with SMI (schizophrenia, schizoaffective and severe affective disorders) was set up by the nurse in conjunction with local care-coordinators and these referred patients invited to enrol in the programme. The care-coordinators were asked to refer patients with SMI who may benefit from inclusion in the WSP. The cohort thus should be considered an SMI cohort where concern existed regarding their physical health monitoring or status. A few patients self-referred and referrals were also accepted from local psychiatrists. Data was not available on the patients declining enrolment in the programme nor on the precise diagnoses of the enrolled patients. Local general practitioners were informed of the service and the majority signed up to shared care agreements thus facilitating communication of information regarding the physical health of these patients. For those patients without a GP, the nurse adviser would work with the local community mental health team (CMHT) to get the patient registered. For those patients who required primary care intervention, who had minimal contact with their GP, the nurse adviser would facilitate their attendance at the GP surgery for an appointment.

### Details of the WSP

Each enrollee was asked to attend for a minimum of six consultations over a maximum of 2 years. The WSP was divided into key steps.

Step 1: A register of SMI patients was generated and these patients were invited to attend the Well-being Support Programme. The nurse adviser set up a weight management and physical activity group in the setting of their choice (usually the community mental health team base or the local GP practice) that continued throughout the 2 years of the programme.

Step 2: First consultations involved a basic physical health check (BP, pulse, weight and height), assessment of lifestyle (diet, physical activity, smoking rates) and medication side effects (Liverpool University Side Effect Rating Scale, LUNSERS) (14).

The nurse advisers were trained in assessing physical health risk factors. They rated the responses to a series of questions regarding type of diet and amount and quality of exercise and the presence of previous physical health checks.

Patients were asked as to whether they had been having regular physical health checks undertaken by their general practitioner, psychiatrist or other. The time-scale utilised was at any time during the previous 12 months. The question asked was ‘Have you been receiving regular physical health checks or advice? For example blood pressure, pulse monitoring or checks for diabetes?’

Diet and activity were assessed by self-report using Likert type scales with categorical responses:

(A) Diet had three categories:

- Unhealthy
- Average
- Healthy

The nurse would rate the patient's diet based on how they described the foods and fluids they had consumed in the last few days and asking them if this fairly represented their eating habits. These ratings were based on how the person described their diet in terms of eating pattern, food choice and cooking methods. Nurses would initially ask patients to rate their own diet. Patients with unhealthy diets would often consider their diet to be healthy. This helped as a starting point in terms of educational needs around healthy eating.

- Healthy diet was assessed using established principles of nutrition (regular meals and drinks, daily consumption of calories, low sugar, low fat diet and high in varied fibre types). Examples of such advice include ‘Wired for Health’, a UK government sponsored education programme (15). An example might be a diet consisting of five portions of fruit and vegetables, a third carbohydrates and the final-third an admixture of protein, dairy foods and some fats (15). The diet should be generally low in sugar, low in fat and high in fibre. Calorie intake would approximate to < 2000 kcal (females) and < 2500 kcal (males). Meals would be eaten three times/day and there would not be a dominance of ‘fast food’.
- Average diet probably had the most variables – this could be higher in fat, regular meals but most likely overeating, probably over eating fats and sugars and under eating fruits and vegetables. The most common example would include excess calorie intake on 1–2 days each week and ‘fast-food’ as predominant meals 1–2 days each week.
- Unhealthy – irregular eating pattern, poor nutritional content, excess of sweets and crisps, and many high fat foods (fast food). These diets would consist often of predominant meals not cooked at home but high-fat ‘fast food’ and an absence of fresh fruit and vegetables

(B) Activity, which resulted in sweating and slight breathlessness, was designated as exercise. Specific questions were asked regarding walking, running, game sports, swimming, gym and other. Duration was recorded in minutes. The nurses rated the amount of such activity per week.

Step 3: The second consultation covered a discussion of the results of the LUNSERs, blood tests (random blood glucose, thyroid function, liver function, prolactin, lipid screen and any other measures at the request of the treating clinician).

Step 4: An individual patient could undergo any one or more of the following as required:

- Referral to a group for weight management or physical activity;
- Referral to GP for further physical health care (via psychiatrist);
- Referral to specialist for further physical health care (via psychiatrist);
- Change of medication (by psychiatrist).

Thus in addition to these individual consultations, the nurse adviser provided access to Healthy Living and/or Weight Management groups and physical activity groups. The structure of the groups varied between different sites. Some nurses offered separate weight management groups, in other areas, these were included as part of the healthy living group. The groups were mostly run by the same nurse adviser, however, in some areas appropriate groups already in existence were used.

### Statistical analysis

As the data is derived from an audit, descriptive data is presented to include mean values and standard deviations where appropriate. Spearman's rho correlation coefficient was used to assess relationships between different variables. Statistical analyses were done using spss v. 12 for Windows (SPSS Inc., Chicago, IL, USA).

## Results

A total of 966 patients enrolled in the programme, 10 patients were excluded from the analysis because of unreliable data. The demographic breakdown of the remaining 956 patients can be seen in Table 1. The average age of the cohort was 46.6, however, 42% were aged over 49 years.

**Table 1:** Baseline demographics

National audit	Male (%)	Female (%)	Total numbers (%)
Total numbers	489 (51)	467 (49)	956 (100)
Age < 30	49 (10.0)	26 (5.5)	75 (7.9)
30–39	117 (23.9)	97 (20.7)	214 (22.4)
40–49	135 (27.6)	133 (28.4)	268 (28)
> 49	188 (38.5)	211 (45.4)	399 (41.7)
**Ethnicity**			
White Caucasian			826 (86.4)
White European			5 (0.5)
Black Caribbean			26 (2.7)
Black African			19 (2.0)
Black British			32 (3.4)
Mixed black/white			5 (0.5)
Southeast Asian			40 (4.2)
Far East Asian			3 (0.3)

**Figure 1:**
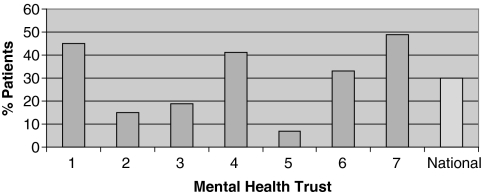
Physical health checks at baseline

Prior to entering the programme, on average only 31% of the patients had undergone regular physical health checks during the previous year, although there was a wide variation across the seven geographical locations that ranged from 7% to 49% (Figure 1). In our cohort of 966 patients, 92% received individual weight management as part of the routine nurse consultations, 51% were also referred to a weight management group and 47% to a physical activity group.

Mean BMI was 31 and 81% had a BMI > 25, 49% had a BMI > 30 indicating obesity and 24% had a BMI > 35 indicating morbid obesity (Table 2). Significantly more females had BMI > 35 than males (30% vs. 18%; p < 0.01).

**Table 2:** BMI at baseline

		All (*n* = 893)		Female (*n* = 438)		Male (*n* = 455)	
	BMI	%	*n*	%	*n*	%	*n*
Underweight	< 18	0	0	0	0	0	0
Normal	18–25	19	174	18	79	21	95
Overweight	26–30	32	283	28	124	35	159
Obese (class 1)	31–35	25	223	24	105	26	118
Obese (class 2)	> 35	24	213	30	130	18	83

Blood pressure measurements were available for 890 of the sample. Mean blood pressure was 132/82 in this group (Table 3). Slightly <50% of the group had a normal blood pressure. 21% were prehypertensive, 29% had grade 1 or more hypertension and 2.0% had severe hypertension (Table 4).

**Table 4:** Blood pressure according to British Hypertension Society guidelines 2004

Category	Systolic blood pressure (mmHg)	Diastolic blood pressure (mmHg)	Count (%)
Normal BP	< 130	< 85	442 (49.7)
High-normal BP	130–139	85–89	189 (21.2)
Grade 1 hypertension (mild)	140–159	90–99	196 (22.0)
Grade 2 hypertension (moderate)	160–179	100–109	46 (5.2)
Grade 3 hypertension (severe)	≥ 180	≥ 110	17 (1.9)
Isolated systolic hypertension (grade 1)	140–159	< 90	111 (12.5)
Isolated systolic hypertension (grade 2)	≥ 160	< 90	20 (2.2)

**Table 3:** Mean findings at baseline

Baseline	Mean (SD)	Range
Age, *n* = 956	46	18–77
BMI, *n* = 893	31.2 (6.7)	18–59
Cigarettes, *n* = 956	13.4 (15.0)	Range 0–60
Alcohol (units), *n* = 956	7.2 (16.8)	0–168
BP *n* = 890 DBP SBP	132/82 82 (12) 132 (18)	DBP 50–131 SBP 75–224
Activity (min), *n* = 956	57 (61.1)	0–180
Diet, *n* = 956	36.4 (25.1)	0–100
Random glucose (mmol/l), *n* = 714	6.1 (2.8)	2.5–28.9
HbA1c, *n* = 188	5.7 (1.5)	4.1–13.9
Triglyceride, *n* = 209 (mmol/l)	2.3 (1.3)	0.6–6.4
HDL cholesterol, *n* = 209 (mmol/l)	1.2 (0.5)	0.57–4.7
AST, *n* = 345 (IU/l)	25.1 (13.7)	9–151
ALT, *n* = 828 (IU/l)	29.3 (18.5)	6–131
Alkaline phosphatase, *n* = 832 (IU/l)	118.8 (71.7)	13–568
Gamma GT, *n* = 569 (IU/l)	49.6 (71.6)	8–1112
Total bilirubin, *n* = 834 (*μ*mol/l)	8.7 (4.3)	2–40
Albumin, *n* = 834 (g/l)	42.3 (3.5)	26–53
Prolactin, *n* = 535 (IU/l)	548	50–3349
TSH, *n* = 459 (IU/l)	2.3 (3.3)	0.01–54.7
Free T4, *n* = 459 (pmol/l)	15.0 (3.3)	1.5–32.5

The patients in this sample smoked an average of 13.4 cigarettes per day and 50% smoked on a daily basis. Of these 479, 90% smoked more than 10 cigarettes per day and 78% smoked more than 20 cigarettes per day (see Table 3).

Of the patients, 34% did no physical activity at all (Table 5). However, 31% did the recommended 90 min or more of exercise per week. 35% of the patients drank alcohol, with 11% taking more than 21 units/week and 2% taking more than 60 units/week.

**Table 5:** Quality of diet and activity at baseline

**Cigarette smoking**	*n* = 956
Smoker	479 (50%)
Non-smoker	478 (50%)
**Alcohol**	*n* = 956
Alcohol (any consumption)	334 (35%)
No current alcohol	622 (65.1%)
**Activity**	*n* = 956
No regular exercise	358 (37.4%)
**Diet**	*n* = 956
Poor diet	304 (32%)
Moderate diet	502 (52%)
Good diet	150 (16%)

Only 16% of the patients had a good dietary habit (Table 5). 52% had an average score for dietary habit with 32% having a very poor dietary habit.

Blood samples were taken in a non-fasting state. Random glucose measures showed that 12.4% of the sample had a blood glucose > 7.1 mmol/l and 5.5% > 11 mmol/l. 71% had abnormal lipids; the mean triglyceride level was 2.3 mmol/l. Mean HDL cholesterol was 1.2 mmol/l. 31.6% were hyperprolactinaemic (prolactin > 500 IU/l) with 5% having a prolactin > 2000 IU/l. 50% had abnormal liver enzymes (50% raised alkaline phosphatase, 19% raised gamma-glutamyl transpeptidase (GT), 3% raised aspartate aminotransferase (AST), and 26% raised alanine transaminase (ALT)). Mean glycosylated haemoglobin (HbA1c) was 5.7% and 35.7% had an HbA1c > 5.7.

Normal ranges were defined as those of each local laboratory who performed the testing. BMI was significantly associated with diastolic blood pressure (*r* = 0.08, p < 0.05). There was a highly significant negative association between BMI and diet (*r* = −0.22, p < 0.001); BMI and level of activity (*r* = −0.17, p < 0.001) and BMI and alcohol intake (*r* = −0.12, p < 0.001). When age was controlled for, BMI continued to be associated with diastolic blood pressure (DBP) (*r* = 0.08, p < 0.05). BMI continued to be negatively associated with diet (*r* = −0.22, p < 0.001), alcohol (*r* = −0.11, p < 0.01) and level of activity (*r* = −0.17, p < 0.001). Hence, being overweight was associated with having a higher blood pressure, a poorer diet and less exercise. It is of note that activity was also negatively associated with DBP (*r* = −0.08, p < 0.05) and heart rate (*r* = −0.07, p < 0.05) but positively associated with diet (*r* = 0.16, p < 0.001). Thus more active individuals had lower blood pressure, lower heart rates and better diets.

## Discussion

A total of 966 patients with SMI enrolled in the Well-being Support Programme. Prior to entering the programme, only 31% had received any kind of regular physical health checks and this figure was highly variable regionally. Our data has shown a high prevalence of potentially modifiable physical health risk factors in this group of patients. One of our aims was to establish the likely physical health state of a routine population of SMI patients in the UK. The relationships between the risk factors were similar to those we would expect to see in the general population, however, the effects of the progression of the illness, lifestyle, poor dietary choices and poverty combined with psychotropic treatments may substantially increase their risk of CVD in the absence of any intervention (16).

The WSP was designed as an intervention that may benefit SMI patients regardless of diagnosis and medication status. Data was thus not collected on specific diagnosis and medication and this is a significant limitation of the data and its extrapolation. Any role of medication or diagnosis on these measured baseline parameters is outside of the scope of this audit.

Most of the group were overweight, with 49% being in the obese range and 24% of the group were severely obese. Females in particular had a significantly increased propensity for BMI > 30 compared with males (30% vs. 18%; p < 0.01). Obesity is one of the traditional risk factors for CVD (16). In this group, BMI was significantly correlated to poor diet, lack of exercise and raised DBP. This is an important correlation in terms of potentially being able to address these significant CVD risk factors through the WSP and similar programmes. Paton found that weight had been recorded in routine practice in 19% of SMI patients (9). In addition to obesity other major CVD risk factors were prevalent. Using the British Hypertension Society 2004 guidelines (17) only 50% of this cohort was found to have a normal blood pressure. 29% had hypertension and an additional 21% had high-normal (prehypertension) blood pressure that might be reduced by lifestyle changes. The CATIE study of schizophrenia patients with fasting laboratory parameters (693 patients) report a higher prevalence of hypertension (47%) in categorical terms (18) yet lower mean BP values than in our audit. CATIE also reports BP data in comparison with the USA general population (NHANES). The clear findings in CATIE were that female schizophrenia patients have a particular propensity for BP elevation compared with a matched control population.

Dyslipidaemia was frequent in this group (71%) and rates were consistent with those found in other SMI populations. Paton reported hyperlipidaemia was present in 68% of her sample of SMI patients (9). Blood samples, however, were not fasting samples. Fasting lipids provide a greater degree of accuracy and non-fasting triglyceride values are essentially invalid. CATIE reports that hyperlipidaemia criteria for the metabolic syndrome were met in 42–50% for triglycerides and 49–63% for HDL in fasting samples (18). Figures were substantially lower in the control populations in NHANES indicating that hyperlipidaemia is more prevalent in an SMI population. This was a particular finding also in females who had twice the likelihood of hyperlipidaemia.

We found that 12.4% of the sample had a random glucose test > 7.1 nmol/l. Of these, 5.5% had glucose levels > 11 nmol/l indicating that they had diabetes. A single fasting glucose sample does not provide greater sensitivity or specificity to diagnose diabetes than a non-fasting sample (19). The gold standard for testing can be considered the oral glucose tolerance test (19). For those with blood glucose levels between 7.1 and 10.9 mmol/l, an oral glucose tolerance test may be considered to confirm whether or not they have diabetes. Although the percentage with frank diabetes is only 5.5%, the 6.9% with prediabetes are at significantly higher risk of subsequently developing increased cardiovascular mortality or morbidity even without the onset of frank diabetes (20,21). Recent advice emphasises the need for effective lifestyle interventions in this group (22). This clearly has major health implications, particularly as few of the patients were known to be diabetic prior to entering the programme. The diagnosis of diabetes is often missed in the SMI population and rates of known glucose abnormalities (prediabetes in particular) rise substantially with glucose testing (23–26). Glucose testing thus identifies a cohort of SMI patients with prediabetes for whom lifestyle interventions are appropriate (22).

It is well known that patients with long-term mental health problems smoke significant amounts of cigarettes (4,7,8). In this programme, 50% were regular smokers. The relatively low levels in this study compared with other studies probably reflect the fact that this was predominantly a community sample rather than an inpatient sample. Despite smoking less than an inpatient sample, the rates are high in this group. Of those that did smoke, most smoked over 20 cigarettes per day. Smoking is a well-known risk factor for CVD.

A large proportion of the patients in this programme had a poor diet and this was associated with high BMI, lack of exercise and low self esteem. Improving dietary habits might not only help to reduce weight but also increase self esteem.

Unsurprisingly, there were high rates of hyperprolactinaemia in the sample. Many datasets on prolactin report only mean data from a population rather than categorical data. When categorical data are looked at, at least 38–69% of an SMI population might be expected to have hyperprolactinaemia, with factors such as the number of females in the cohort and the antipsychotics prescribed being important (27–30). Of concern is that 5% had prolactin levels over 2000 IU/l, which many clinicians would wish to investigate further to exclude a prolactin-secreting tumour (29–31). As these patients were not receiving regular health checks prior to the WSP, these findings were missed. A proportion of this group were referred for CT or MRI scans to exclude brain pathology. This finding indicates a need for more regular prolactin screening in people who take antipsychotic drugs. In addition, hyperprolactinaemia may be the most important aetiological factor in the subsequent development of osteoporosis and hip fractures (32–34).

Almost half the sample had some degree of liver function abnormality, which indicates a need to ascertain the reason for these abnormalities. This was a cholestatic picture which is found in overweight, but can also be a side effect of antipsychotic medication. This finding needs further investigation.

Despite primary care incentives to improve detection and monitoring of physical disease, generally the people entering this study were unlikely to have been receiving regular physical health checks. One of the problems preventing monitoring is confusion over whose responsibility it is to do the monitoring. The UK National Service Framework and NICE guidelines indicate that primary care should be responsible for this monitoring and the new GP contract is designed to facilitate this. However, this audit shows that such monitoring is not taking place on a regular basis. There are likely to be a number of complex reasons for this. A survey by the Mental Health Foundation revealed that many people with psychiatric illness felt unable to approach their GP about physical problems, as they did not believe that they would be taken seriously (35). Apart from this reason, lack of uptake of primary care services may be impacted upon by factors outside of the patient group. Kendrick postulated that GPs may find patients with SMI difficult to communicate with (10). Salmon et al. suggested that patients with SMI may be harder for the system to ‘process’ because they are chaotic or difficult (36). Although mental health clinicians are not adequately trained in chronic physical health assessment and physical health promotion to be able to provide effective primary care services, in many cases they may have more regular contact with SMI clients. Therefore, mental health clinicians could provide basic physical health monitoring for cardiovascular risk factors and facilitate patients with SMI attending primary care services. The Well-being Support Programme has developed a training programme to improve such skills in mental health staff.

These data have shown that in the UK the vast majority of SMI patients in this chosen cohort have easily recognisable risk factors for CVD (hypertension, hyperlipidaemia, obesity, smoking, poor dietary choices and low exercise levels). Mortality and morbidity in these patients will not begin to decline unless these issues are appropriately addressed. The rates of physical health risk factors in this population are extremely high and are a significant public health issue which needs to be addressed as a matter of urgency. This group of patients do not have the same access to physical health care as the rest of the population. The reason for this inequity of service needs to be investigated. Evidence should be sought to ascertain if lifestyle interventions can modify known physical disease risk factors in the severely mentally ill.

## Limitations

The absence of a population-based control group is a significant limitation in the interpretation of these data and we cannot be certain as to the extent of ‘hidden’ physical illness and cardiovascular risk factors present comparatively. Despite full training and an audit tool into which collected data blood sampling was not complete for all patients. The initial aim was to collect fasting samples pragmatically, however, this is not possible easily in an outpatient SMI cohort with the various limitations including timing of appointments and laboratory schedules. Random sampling was thus undertaken. Any interpretation of these data are limited to the chosen cohort as they cannot be necessarily considered to be a randomly chosen cohort, chosen however because of some concern over their physical health monitoring. We also were not able to collect the precise diagnosis for each patient nor their medication schedules. We chose to use the concept of SMI as an overarching clinical diagnosis as the concept of SMI is a clinical one and is included in many government policy documents and other bodies. Most clinicians understand SMI to include the severe psychoses and severe affective disorders. This term is used in the disability rights commission report ‘Equal Treatment, Closing the Gap’ (37). This report also used the broadest definition on mental health problems to outline the health inequalities between people with mental health problems and the general public. Our paper also uses a broad clinical definition of SMI but this lack of precision over the exact ICD-10 diagnosis should be regarded as a limitation in these data. A final limitation is the absence of data on medications taken by the subjects as these data may have been helpful in further interpreting the baseline health data of the subjects.

## Supplementary material

The following supplementary material is available for this article:

**Slide Show S1.** A Well-being programme in Severe Mental Illness. Baseline findings in a UK cohort.

This material is available as part of the online article from: http://www.blackwell-synergy.com/
