# Supplementary material for: A well-being programme in severe mental illness. Baseline findings in a UK cohort
Source: Int J Clin Pract. 2007 Dec;61(12):1971–8. doi: 10.1111/j.1742-1241.2007.01605.x (PMC2779991; doi:10.1111/j.1742-1241.2007.01605.x)
Supplement: Supplementary file 1 [file ijcp0061-1971-SD1.ppt]

## Slide 1
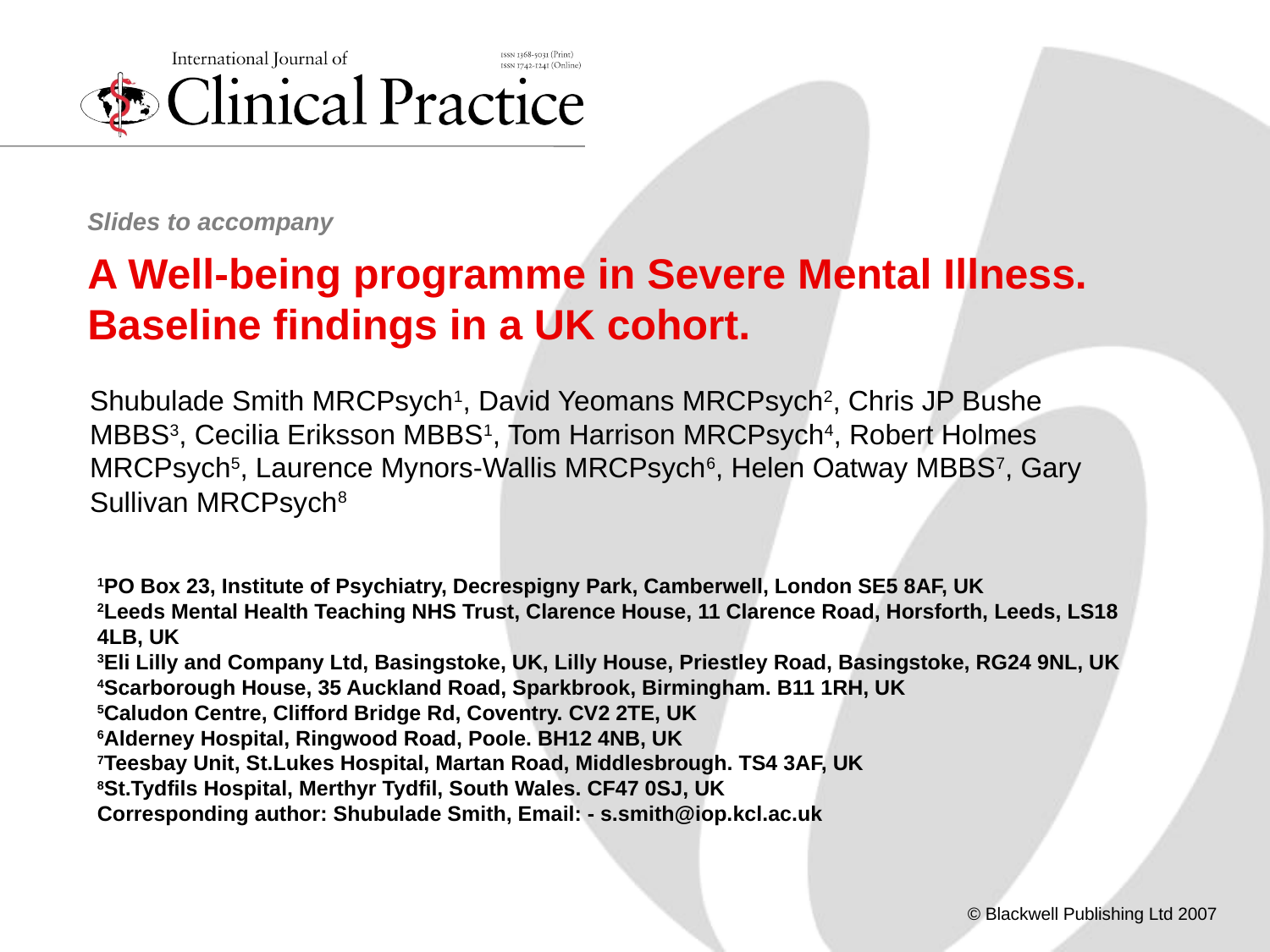

# A Well-being programme in Severe Mental Illness. Baseline findings in a UK cohort.
Shubulade Smith MRCPsych1, David Yeomans MRCPsych2, Chris JP Bushe MBBS3, Cecilia Eriksson MBBS1, Tom Harrison MRCPsych4, Robert Holmes MRCPsych5, Laurence Mynors-Wallis MRCPsych6, Helen Oatway MBBS7, Gary Sullivan MRCPsych8
1PO Box 23, Institute of Psychiatry, Decrespigny Park, Camberwell, London SE5 8AF, UK
2Leeds Mental Health Teaching NHS Trust, Clarence House, 11 Clarence Road, Horsforth, Leeds, LS18 4LB, UK
3Eli Lilly and Company Ltd, Basingstoke, UK, Lilly House, Priestley Road, Basingstoke, RG24 9NL, UK
4Scarborough House, 35 Auckland Road, Sparkbrook, Birmingham. B11 1RH, UK
5Caludon Centre, Clifford Bridge Rd, Coventry. CV2 2TE, UK
6Alderney Hospital, Ringwood Road, Poole. BH12 4NB, UK
7Teesbay Unit, St.Lukes Hospital, Martan Road, Middlesbrough. TS4 3AF, UK
8St.Tydfils Hospital, Merthyr Tydfil, South Wales. CF47 0SJ, UK
Corresponding author: Shubulade Smith, Email: - s.smith@iop.kcl.ac.uk

## Slide 2
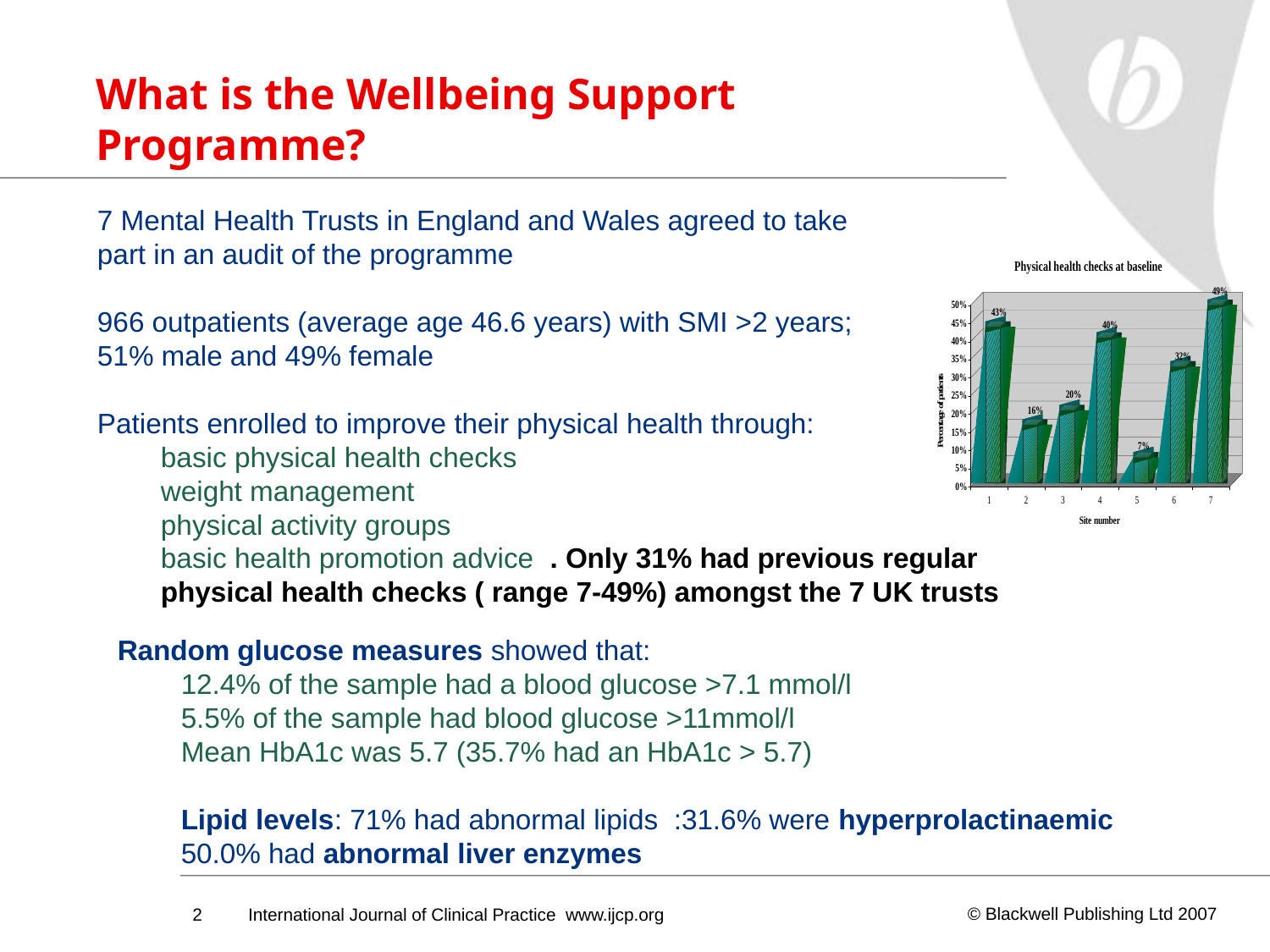

# What is the Wellbeing Support Programme?
7 Mental Health Trusts in England and Wales agreed to take
part in an audit of the programme
966 outpatients (average age 46.6 years) with SMI >2 years;
51% male and 49% female
Patients enrolled to improve their physical health through:
basic physical health checks
weight management
physical activity groups
basic health promotion advice . Only 31% had previous regular physical health checks ( range 7-49%) amongst the 7 UK trusts
Random glucose measures showed that:
12.4% of the sample had a blood glucose >7.1 mmol/l
5.5% of the sample had blood glucose >11mmol/l
Mean HbA1c was 5.7 (35.7% had an HbA1c > 5.7)
Lipid levels: 71% had abnormal lipids :31.6% were hyperprolactinaemic
50.0% had abnormal liver enzymes
International Journal of Clinical Practice www.ijcp.org
2

## Slide 3
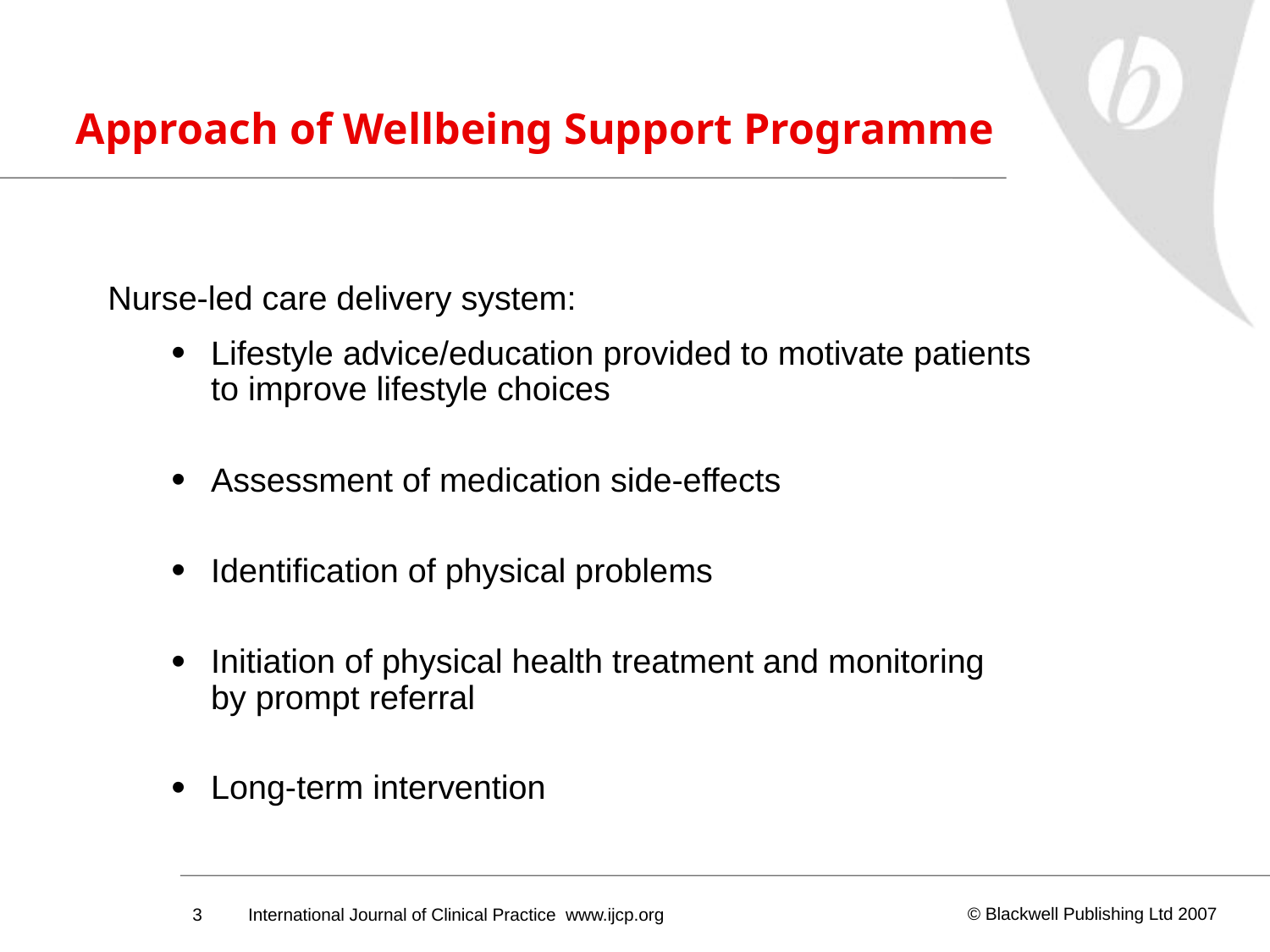

Approach of Wellbeing Support Programme
Nurse-led care delivery system:
Lifestyle advice/education provided to motivate patients
to improve lifestyle choices
Assessment of medication side-effects
Identification of physical problems
Initiation of physical health treatment and monitoring
by prompt referral
Long-term intervention
International Journal of Clinical Practice www.ijcp.org
3

## Slide 4
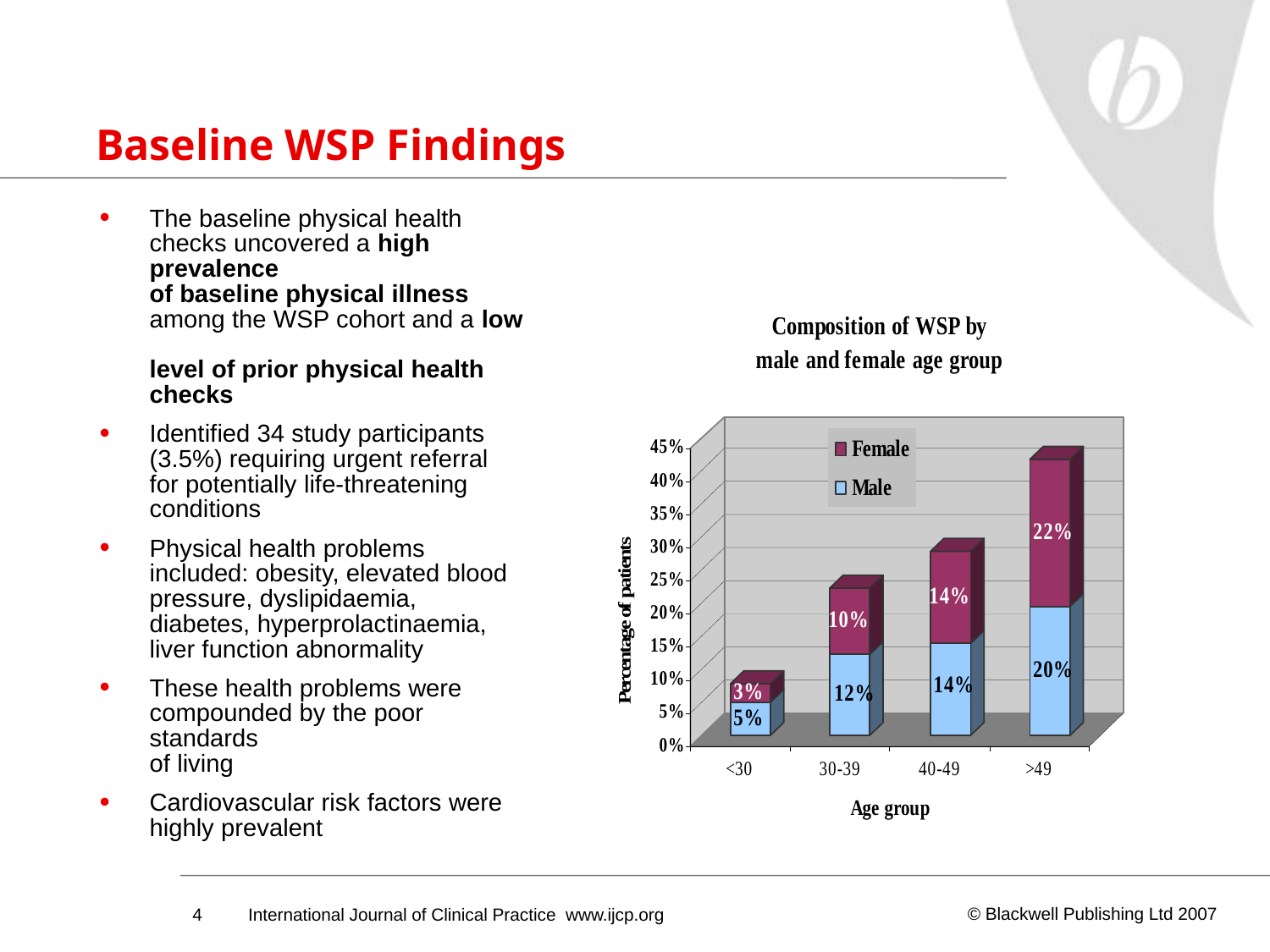

# Baseline WSP Findings
The baseline physical health checks uncovered a high prevalence
of baseline physical illness among the WSP cohort and a low
level of prior physical health checks
Identified 34 study participants (3.5%) requiring urgent referral for potentially life-threatening conditions
Physical health problems included: obesity, elevated blood pressure, dyslipidaemia, diabetes, hyperprolactinaemia, liver function abnormality
These health problems were compounded by the poor standards
of living
Cardiovascular risk factors were highly prevalent
International Journal of Clinical Practice www.ijcp.org
4

## Slide 5
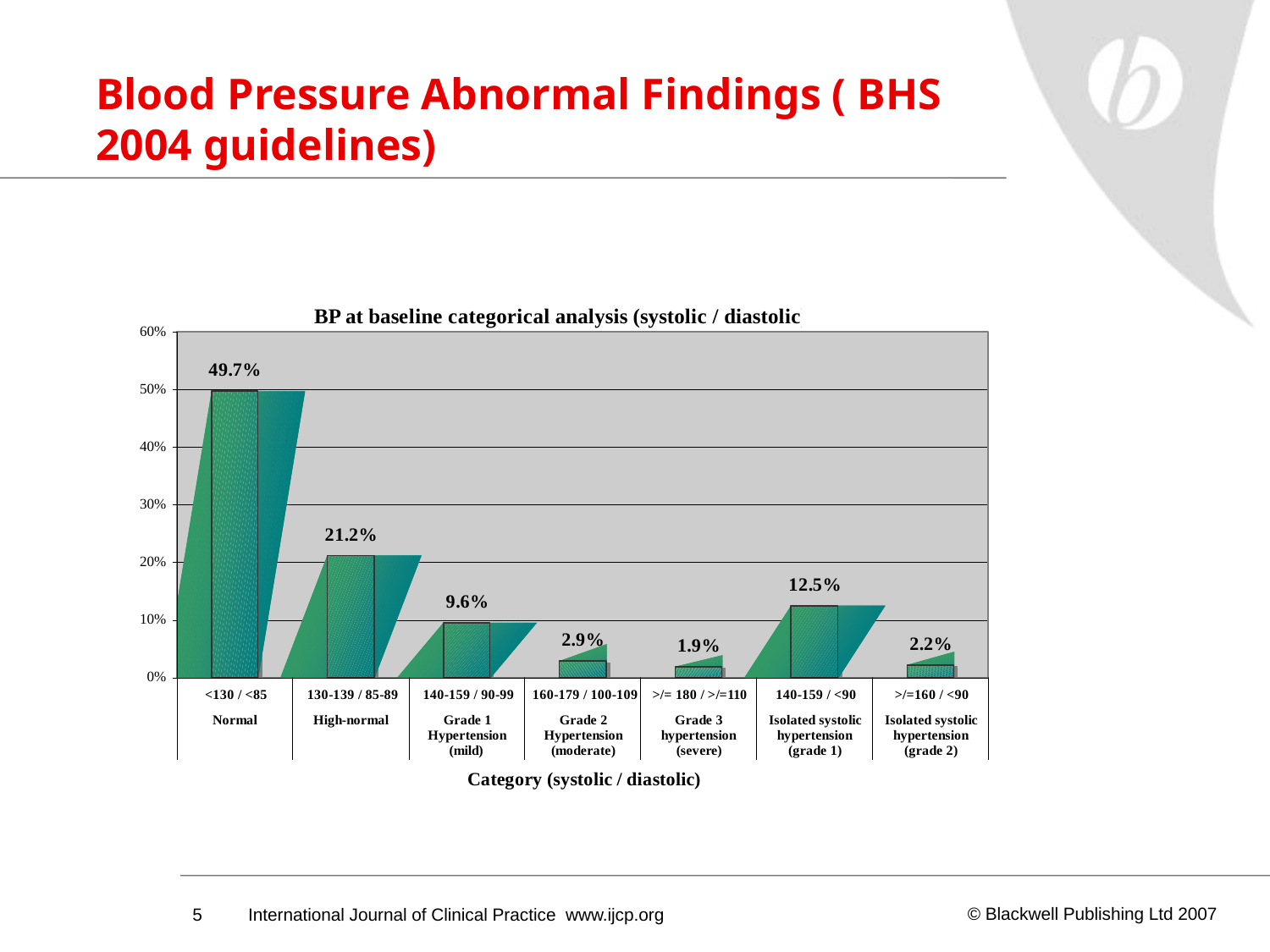

# Blood Pressure Abnormal Findings ( BHS 2004 guidelines)
International Journal of Clinical Practice www.ijcp.org
5

## Slide 6
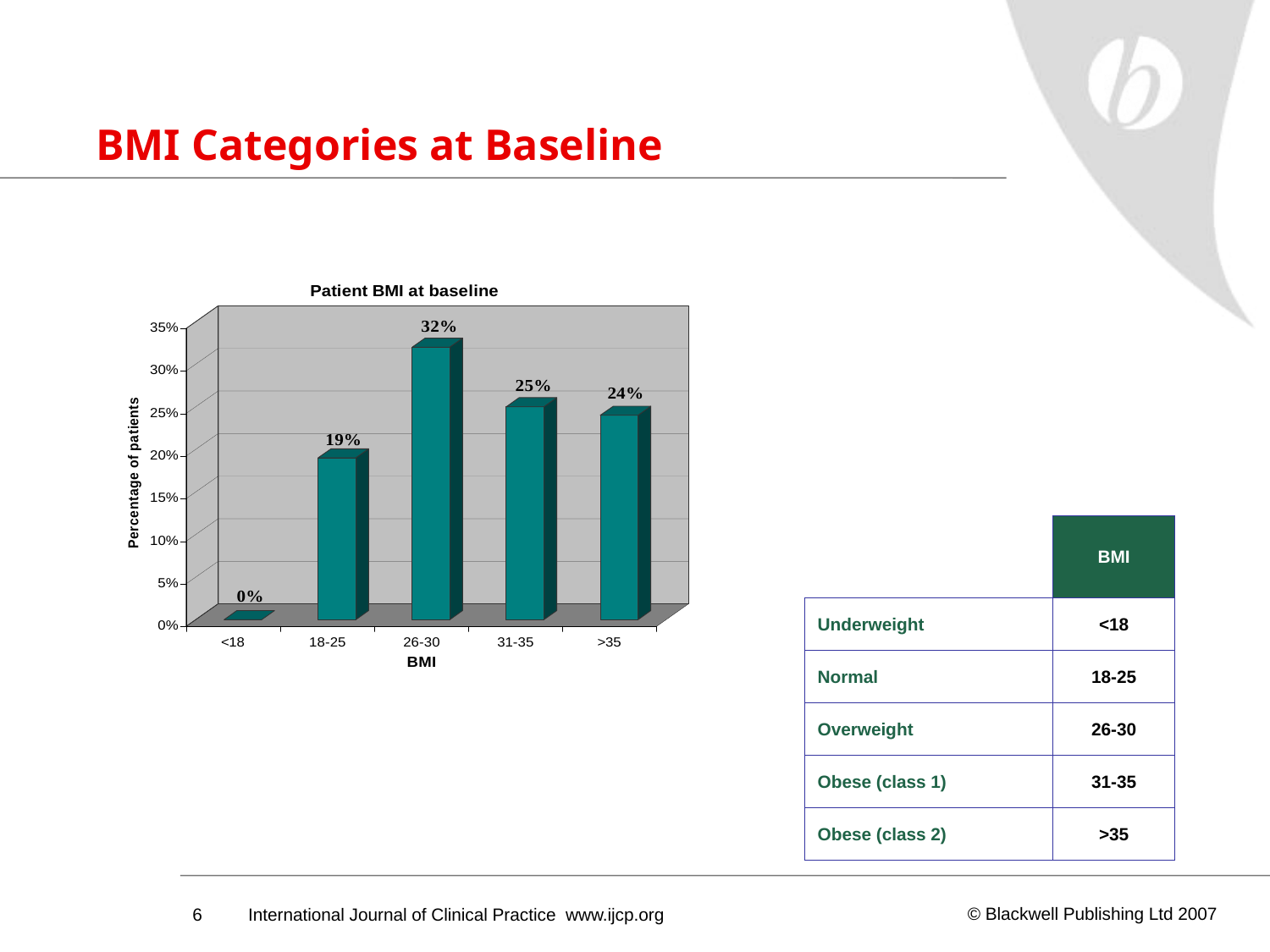

# BMI Categories at Baseline
| | BMI |
| --- | --- |
| Underweight | <18 |
| Normal | 18-25 |
| Overweight | 26-30 |
| Obese (class 1) | 31-35 |
| Obese (class 2) | >35 |
International Journal of Clinical Practice www.ijcp.org
6

## Slide 7
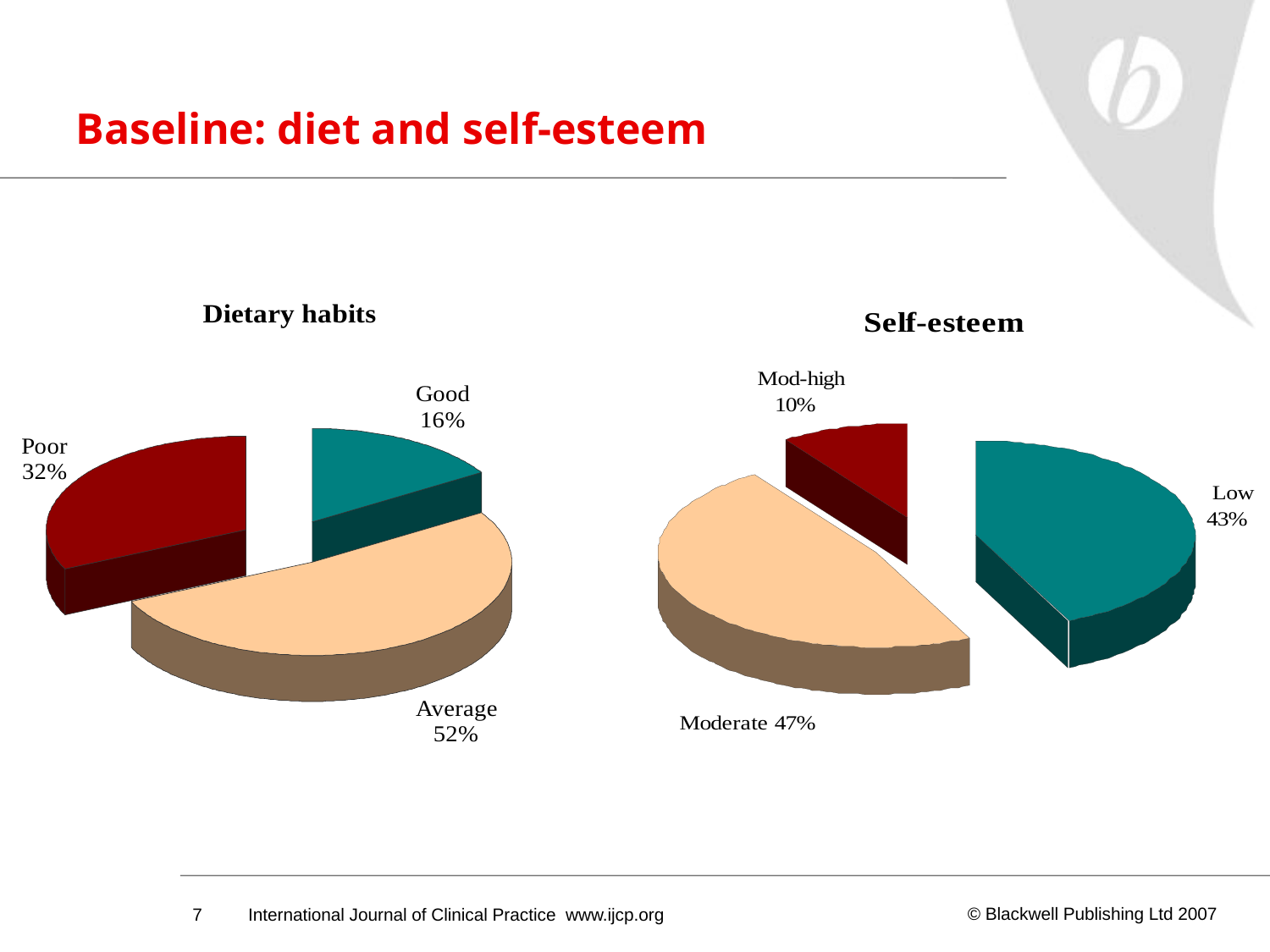

Baseline: diet and self-esteem
International Journal of Clinical Practice www.ijcp.org
7

## Slide 8
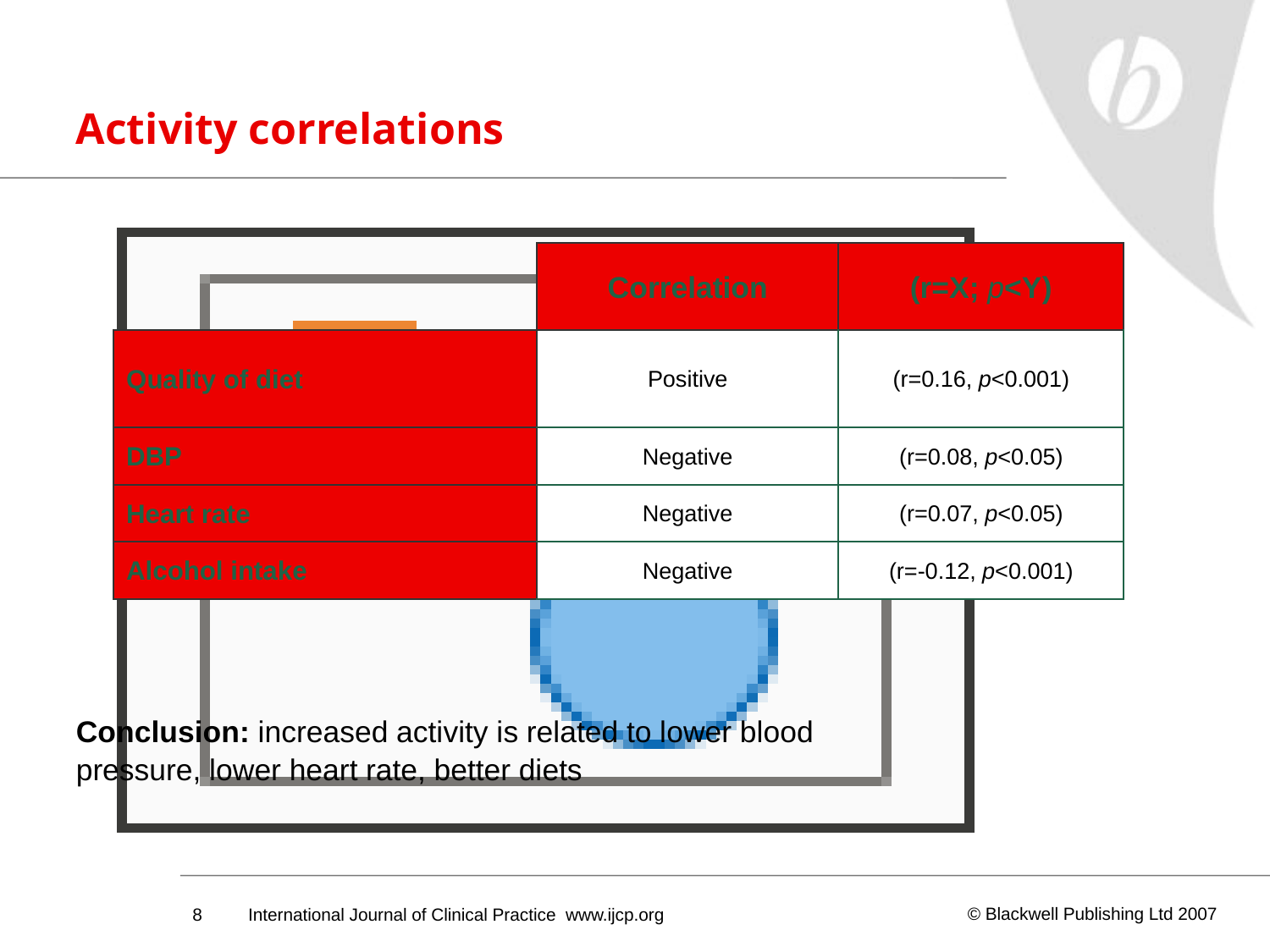

Activity correlations
| | Correlation | (r=X; p<Y) |
| --- | --- | --- |
| Quality of diet | Positive | (r=0.16, p<0.001) |
| DBP | Negative | (r=0.08, p<0.05) |
| Heart rate | Negative | (r=0.07, p<0.05) |
| Alcohol intake | Negative | (r=-0.12, p<0.001) |
Conclusion: increased activity is related to lower blood
pressure, lower heart rate, better diets
International Journal of Clinical Practice www.ijcp.org
8
